# Supplementary material for: RBI: a novel algorithm for regulatory-metabolic network model in designing the optimal mutant strain
Source: PeerJ Comput Sci. 2025 May 27;11:e2880. doi: 10.7717/peerj-cs.2880 (PMC12199197; doi:10.7717/peerj-cs.2880)
Supplement: Supplemental Information 1 [file peerj-cs-11-2880-s001.pdf]

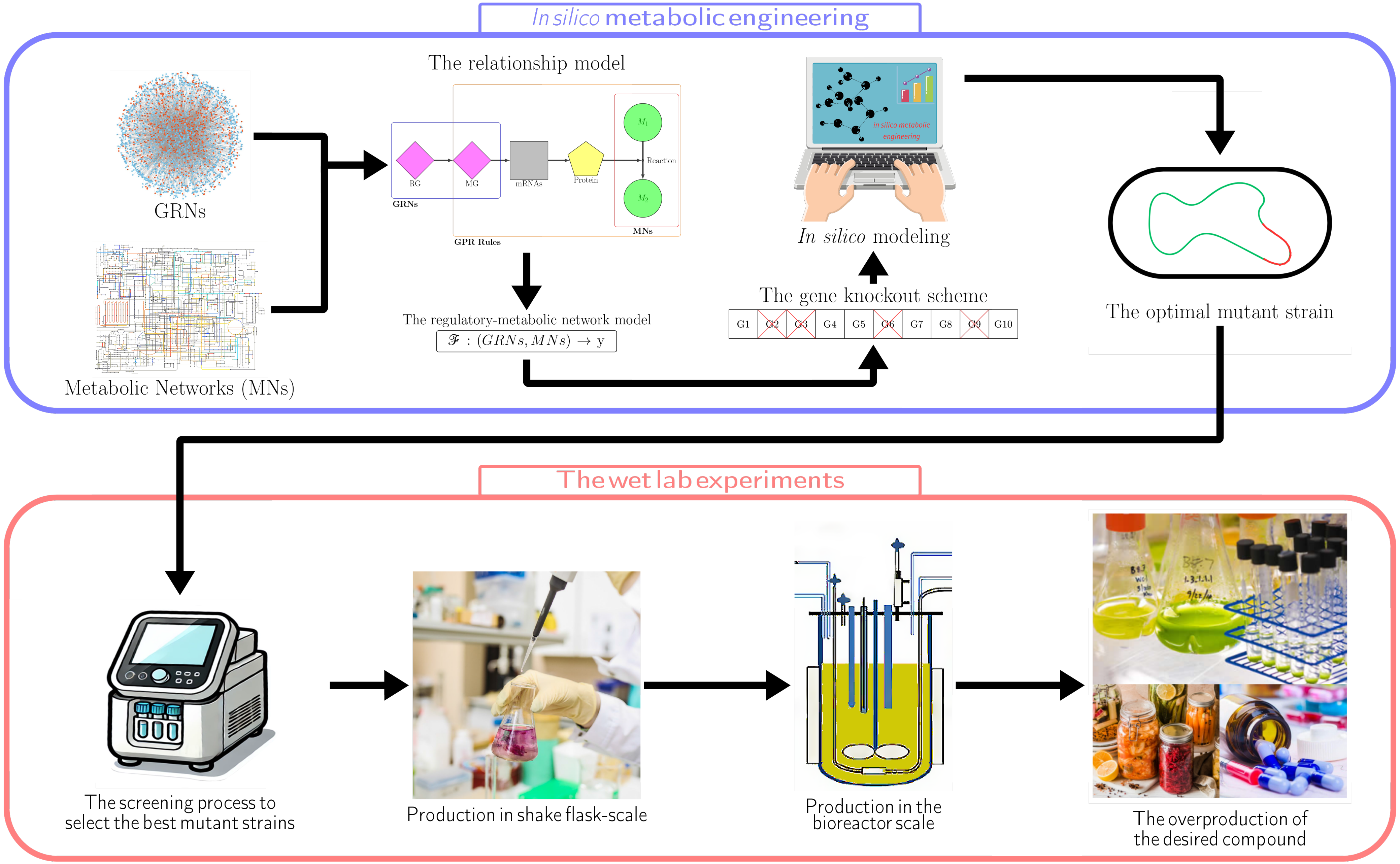

A general procedure *in silico* metabolic engineering for predicting the optimal mutant strain and guiding the wet lab experiments.
