# Supplementary figures and images for: RBI: a novel algorithm for regulatory-metabolic network model in designing the optimal mutant strain
Source: PeerJ Comput Sci. 2025 May 27;11:e2880. doi: 10.7717/peerj-cs.2880 (PMC12199197; doi:10.7717/peerj-cs.2880)

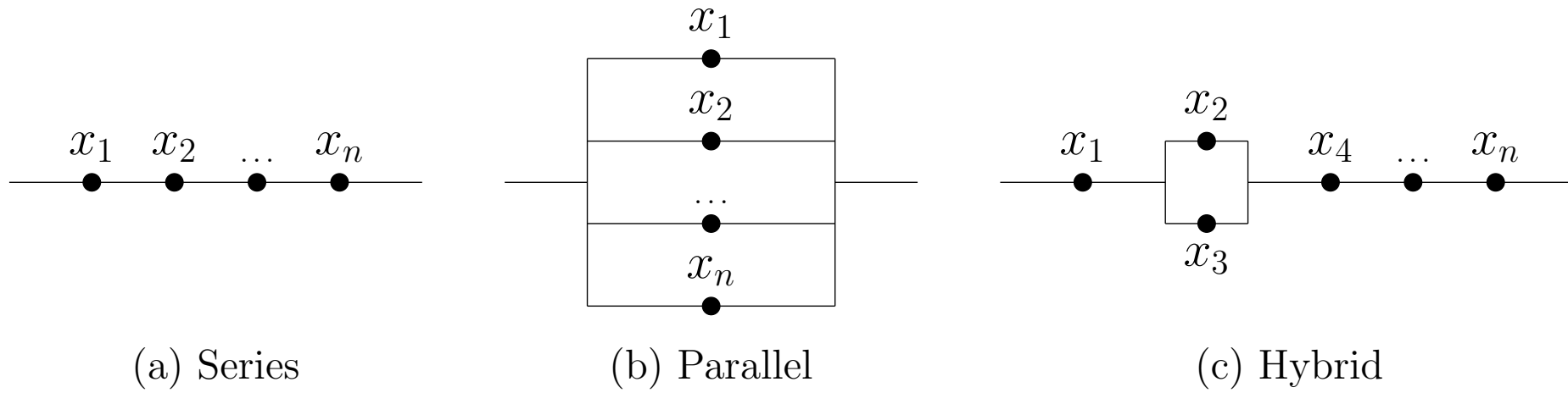

Illustration of series, parallel, and hybrid structures in reliability theory.

Supplement: Supplemental Information 2 [file peerj-cs-11-2880-s002.pdf]

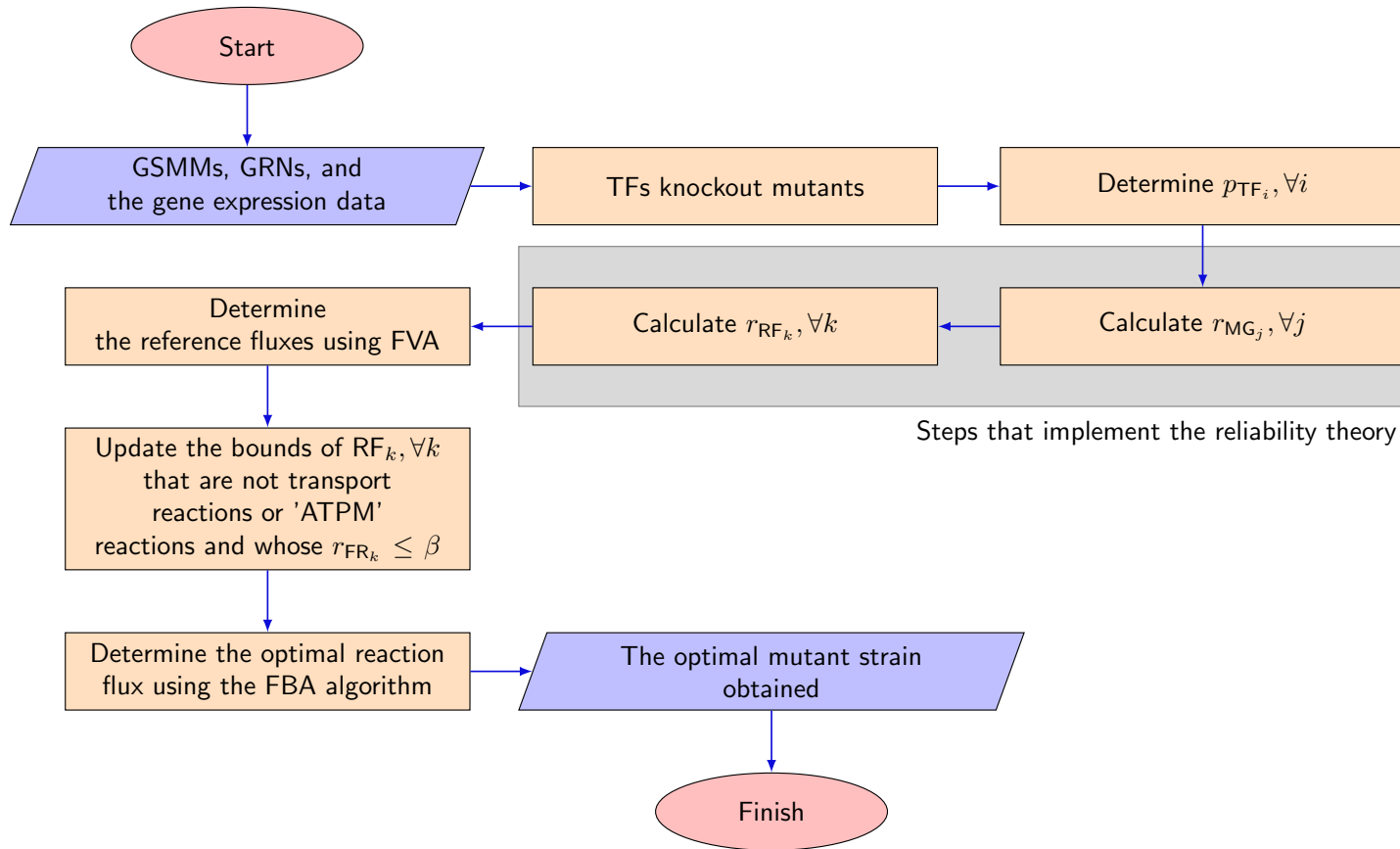

The block diagram of the RBI algorithm

Supplement: Supplemental Information 6 [file peerj-cs-11-2880-s006.pdf]
