# Supplementary material for: RBI: a novel algorithm for regulatory-metabolic network model in designing the optimal mutant strain
Source: PeerJ Comput Sci. 2025 May 27;11:e2880. doi: 10.7717/peerj-cs.2880 (PMC12199197; doi:10.7717/peerj-cs.2880)
Supplement: Supplemental Information 3 [file peerj-cs-11-2880-s003.pdf]

$$aspA = \big(Crp \text{ AND } (\text{ NOT } Fnr)\big) \text{ OR } Fnr$$

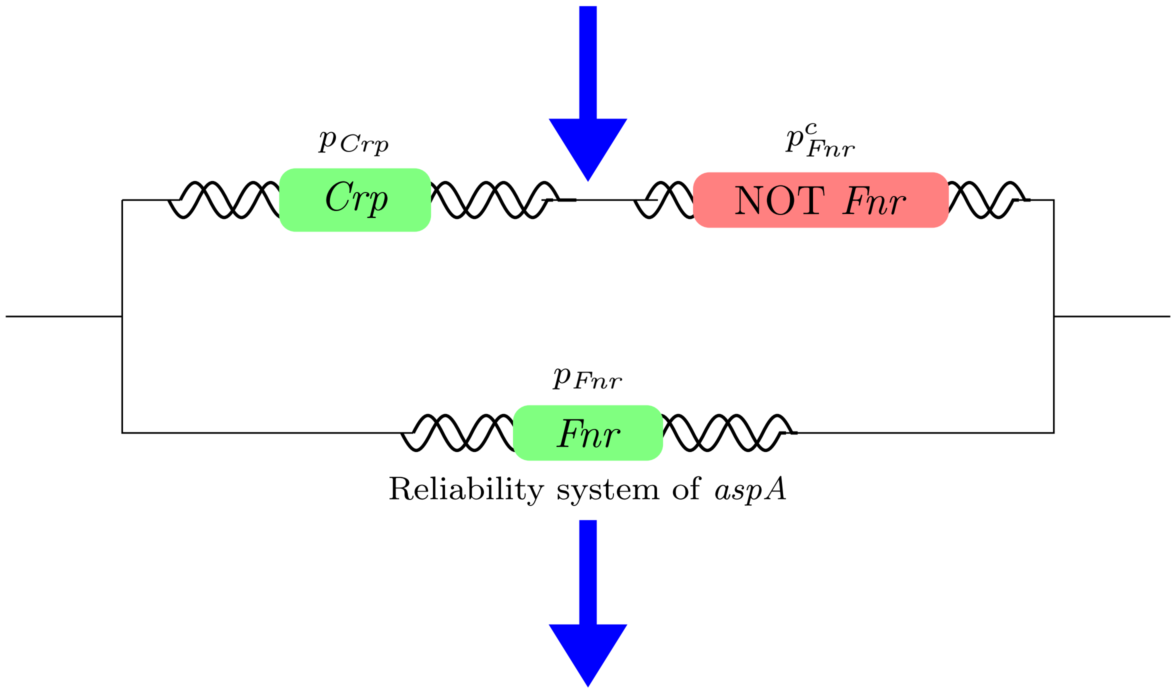

$$r_{aspA} = 1 - (1 - p_{Crp}\, p^c_{Fnr})(1 - p_{Fnr})$$

Illustration of the determining process of the 'aspA' reliability from the empirical GRNs.
