# Supplementary material for: RBI: a novel algorithm for regulatory-metabolic network model in designing the optimal mutant strain
Source: PeerJ Comput Sci. 2025 May 27;11:e2880. doi: 10.7717/peerj-cs.2880 (PMC12199197; doi:10.7717/peerj-cs.2880)
Supplement: Supplemental Information 4 [file peerj-cs-11-2880-s004.pdf]

$$ATPS4r = (AtpF0 \text{ AND } AtpF1) \text{ OR } (AtpF0 \text{ AND } AtpF1 \text{ AND } AtpI)$$

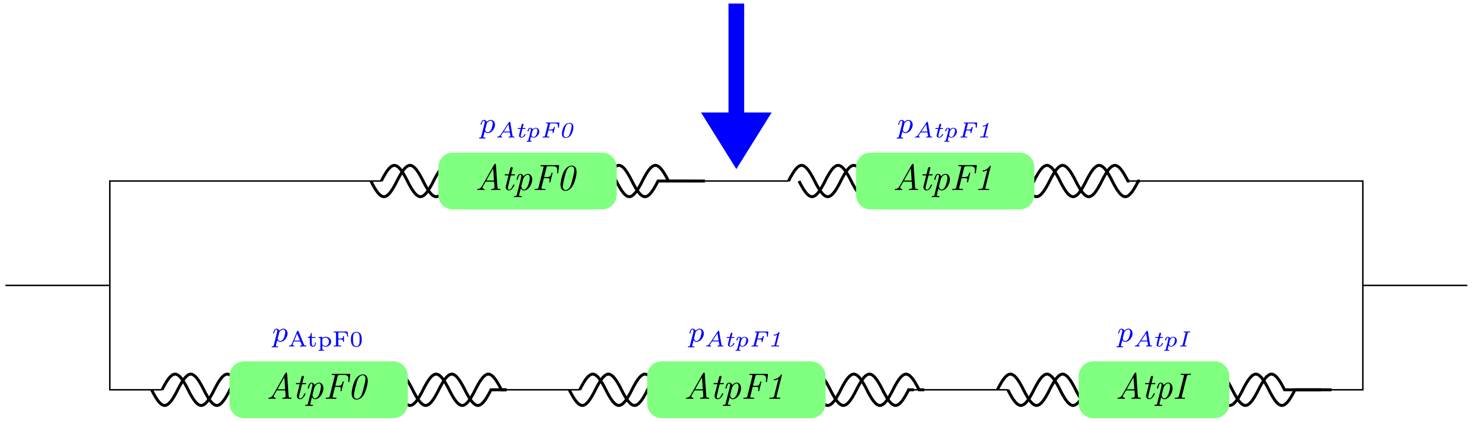

Reliability system of *ATPS4r*

$$r_{ATPS4r} = 1 - (1 - p_{AtpF0}\, p_{AtpF1})(1 - p_{AtpF0}\, p_{AtpF1}\, p_{\text{AtpI}})$$

Illustration of the determining process of the 'ATPS4r' reliability from the GPR rules.
