# Supplementary material for: RBI: a novel algorithm for regulatory-metabolic network model in designing the optimal mutant strain
Source: PeerJ Comput Sci. 2025 May 27;11:e2880. doi: 10.7717/peerj-cs.2880 (PMC12199197; doi:10.7717/peerj-cs.2880)
Supplement: Supplemental Information 5 [file peerj-cs-11-2880-s005.pdf]

The initial probability values of the regulatory gene for each type of RBI

No	Type of RBI	The initial probability values of the ith gene
1	RBI-T1	$p_{\mathrm{TF}_i} = f(\mathrm{TF}_i) = 0.5$
2	RBI-T2	$p_{\mathrm{TF}_i} = f(\mathrm{TF}_i) = \frac{1}{d} \times n(\mathrm{RG}_i = 1)$
3	RBI-T3	$p_{\mathrm{TF}_i} = f(\mathrm{TF}_i) = \frac{1}{2}\left(0.5 + \frac{1}{d} \times n(\mathrm{TF}_i = 1)\right)$

Note: Additional information regarding those formulas was included in the RBI steps.
