# Supplementary material for: RBI: a novel algorithm for regulatory-metabolic network model in designing the optimal mutant strain
Source: PeerJ Comput Sci. 2025 May 27;11:e2880. doi: 10.7717/peerj-cs.2880 (PMC12199197; doi:10.7717/peerj-cs.2880)
Supplement: Supplemental Information 7 [file peerj-cs-11-2880-s007.pdf]

The detail of the strain models used

Strain	The number of			Metabolite	References
	Reactions	Metabolites	Genes		
iAF1260	2382	1668	1261	Biomass, Indole	Niu et al. (2021)
Yeast7	3498	2220	910	succinate, ethanol, and 2,3-butanediol	Shen et al. (2019)

References

Niu, P., Soto, M. J., Yoon, B.-J., Dougherty, E. R., Alexander, F. J., Blaby, I., and Qian, X. (2021). Trimer: transcription regulation integrated with metabolic regulation. *iScience*, 24(11):103218.

Shen, F., Sun, R., Yao, J., Li, J., Liu, Q., Price, N. D., Liu, C., and Wang, Z. (2019). OptRAM: In-silico strain design via integrative regulatory-metabolic network modeling. *PLOS Computational Biology*, 15(3):e1006835.
