# Supplementary material for: RBI: a novel algorithm for regulatory-metabolic network model in designing the optimal mutant strain
Source: PeerJ Comput Sci. 2025 May 27;11:e2880. doi: 10.7717/peerj-cs.2880 (PMC12199197; doi:10.7717/peerj-cs.2880)
Supplement: Supplemental Information 8 [file peerj-cs-11-2880-s008.pdf]

The optimal parameters of α, β, and γ that were obtained from the optimization process

**Algorithm**	**The microbial strain**					
	E. coli			*S. cerevisiae*		
	α	β	γ	α	β	γ
RBI-T1	-	0.062	0.940	-	0.499	10.000
RBI-T2	0.500	0.116	9.884	0.389	0.258	1.092
RBI-T3	0.500	0.010	7.640	0.372	0.384	8.222

*
