# Supplementary material for: RBI: a novel algorithm for regulatory-metabolic network model in designing the optimal mutant strain
Source: PeerJ Comput Sci. 2025 May 27;11:e2880. doi: 10.7717/peerj-cs.2880 (PMC12199197; doi:10.7717/peerj-cs.2880)
Supplement: Supplemental Information 9 [file peerj-cs-11-2880-s009.pdf]

The performance of the RBI algorithms compared to the existing algorithms in predicting the growth rate for *E. coli* TF-knockout mutants

TF KO	Cond.	Actual	RBI-T1	RBI-T2	RBI-T3	PROM[1]	TRFBA[1]	TRIMER[1]
WT	AER	0.710	0.708	1.032	0.760	0.708	0.563	0.708
arcA	AER	0.686	0.708	1.150	0.760	0.272	0.563	0.610
fnr	AER	0.635	0.702	1.024	0.887	0.526	0.563	0.547
arcA, fnr	AER	0.648	0.702	1.032	0.922	0.272	0.563	0.619
appY	AER	0.636	0.708	1.032	0.760	0.708	0.563	0.708
oxyR	AER	0.637	0.725	1.032	0.844	0.708	0.563	0.708
soxS	AER	0.724	0.708	1.032	0.760	0.707	0.563	0.707
WT	ANA	0.485	0.488	1.182	0.545	0.481	0.407	0.481
arcA	ANA	0.377	0.488	1.490	0.545	0.037	0.355	0.071
fnr	ANA	0.410	0.539	1.182	0.892	0.271	0.353	0.371
arcA, fnr	ANA	0.301	0.539	1.182	0.892	0.037	0.356	0.160
appY	ANA	0.476	0.488	1.182	0.545	0.481	0.354	0.481
ocyR	ANA	0.481	0.506	1.182	0.633	0.481	0.357	0.481
soxS	ANA	0.465	0.488	1.182	0.545	0.479	0.355	0.481
RMSE	-	-	**0.088**	0.634	0.247	0.196	0.100	0.100
PCC	-	-	0.871	-0.737	0.296	0.693	**0.927**	0.906
R-squared	-	-	0.647	0.474	0.473	0.618	0.634	**0.793**
Bias	-	-	0.059	0.589	0.187	-0.107	-0.085	**-0.038**

Note: The unit utilized is mmol/gDCW/hr. Glucose and oxygen uptake rates, under aerobic conditions, are 8.5 and 14.6 mmol/gDCW/hr, respectively (Niu et al., 2021). In anaerobic conditions, they are 20.8 and 0 mmol/gDCW/hr, respectively. [1]The values are provided by Niu et al. (2021). AER and ANA refer to aerobic and anaerobic, respectively.

References

Niu, P., Soto, M. J., Yoon, B.-J., Dougherty, E. R., Alexander, F. J., Blaby, I., and Qian, X. (2021). Trimer: transcription regulation integrated with metabolic regulation. *iScience*, 24(11):103218.

*
