# Supplementary material for: RBI: a novel algorithm for regulatory-metabolic network model in designing the optimal mutant strain
Source: PeerJ Comput Sci. 2025 May 27;11:e2880. doi: 10.7717/peerj-cs.2880 (PMC12199197; doi:10.7717/peerj-cs.2880)
Supplement: Supplemental Information 10 [file peerj-cs-11-2880-s010.pdf]

The performance of the RBI algorithms compared to the existing algorithms in predicting the production rate of Indole for *E. coli* TF-knockout mutants

TF KO	Actual	RBI-T1	RBI-T2	RBI-T3	PROM	TRFBA	TRIMER
Fnr	0.043	0.065	0.065	0.056	0.022	0.010	0.022
soxS	0.039	0.064	0.062	0.069	0.037	0.010	0.036
Crp	0.040	0.065	0.064	0.074	0.000	0.010	0.019
lysR	0.040	0.064	0.062	0.069	0.037	0.010	0.037
fucR	0.039	0.064	0.062	0.069	0.040	0.010	0.040
Mali	0.040	0.064	0.062	0.069	0.040	0.010	0.040
phoB	0.039	0.065	0.062	0.069	0.040	0.010	0.040
cpxR	0.039	0.064	0.062	0.069	0.040	0.010	0.040
tnaA	0.038	0.064	0.062	0.069	0.040	0.010	0.040
tnaB	0.040	0.064	0.062	0.069	0.040	0.010	0.040
RMSE	-	0.025	0.023	0.029	0.014	0.030	**0.010**
PCC	-	0.499	**0.808**	-0.753	-0.447	0.000	-0.661
R-squared	-	**0.500**	**0.500**	0.497	0.480	**0.500**	0.457
Bias	-	0.025	0.023	0.029	-0.006	-0.030	**-0.004**

Note: The unit utilized is mmol/gDCW/hr. Glucose and oxygen uptake rates used are 8.5 and 14.6 mmol/gDCW/hr, respectively (Niu et al., 2021).

References

Niu, P., Soto, M. J., Yoon, B.-J., Dougherty, E. R., Alexander, F. J., Blaby, I., and Qian, X. (2021). Trimer: transcription regulation integrated with metabolic regulation. *iScience*, 24(11):103218.

*
