# Supplementary material for: RBI: a novel algorithm for regulatory-metabolic network model in designing the optimal mutant strain
Source: PeerJ Comput Sci. 2025 May 27;11:e2880. doi: 10.7717/peerj-cs.2880 (PMC12199197; doi:10.7717/peerj-cs.2880)
Supplement: Supplemental Information 11 [file peerj-cs-11-2880-s011.pdf]

The perturbation scheme used in designing the optimal mutant strain of Yeast 7.6

**No**	**Metabolite**	**TFs knock-out**
1	Succinate	GLT1, FPS1, TKL1, MET32, MET4
2	2,3-butanediol	ACS1, COX9, SOL3, IDP2, ADH3, MDH2, CHA4
3	Ethanol	COX4, MDH2, FAA1, ATH1

*
