# Supplementary material for: RBI: a novel algorithm for regulatory-metabolic network model in designing the optimal mutant strain
Source: PeerJ Comput Sci. 2025 May 27;11:e2880. doi: 10.7717/peerj-cs.2880 (PMC12199197; doi:10.7717/peerj-cs.2880)
Supplement: Supplemental Information 12 [file peerj-cs-11-2880-s012.pdf]

The performance of the RBI algorithms compared to the existing algorithms in predicting the flux ranges of succinate (Succ.), 2,3-butanediol (BD), and ethanol (EtOH) productions

Met.	Bio.	Bd.	Lit.[1]	RBI-T1	RBI-T2	RBI-T3	PROM	TRFBA	OptFlux[2]	OptRAM[2]
Succ.	99%	lb	0.100	0.000	0.000	0.000	0.000	0.000	6.780	11.600
		ub	0.430	0.998	0.324	0.978	19.918	19.925	7.460	11.880
	50%	lb	0.000	0.000	0.000	0.000	0.000	0.000	0.030	1.110
		ub	8.200	17.528	4.368	18.553	19.925	19.925	11.910	13.200
BD	99%	lb	5.070	0.000	0.000	0.000	0.000	0.000	0.000	6.860
		ub	6.420	4.368	0.702	6.542	19.917	19.925	5.900	7.200
	50%	lb	0.000	0.000	0.000	0.000	0.000	0.000	0.000	0.000
		ub	9.760	15.386	18.337	15.184	19.925	19.925	8.340	8.540
EtOH	99%	lb	0.000	0.000	28.608	0.000	0.000	0.000	8.130	15.700
		ub	0.220	10.625	30.543	12.687	39.827	39.843	12.800	15.980
	50%	lb	0.000	0.000	0.000	0.000	0.000	0.000	0.000	4.780
		ub	10.900	26.429	35.224	26.206	39.843	39.843	17.200	18.160
RMSE	-	-	-	6.444	14.365	6.784	16.419	16.423	**5.772**	8.492
PCC	-	-	-	**0.819**	0.305	0.811	0.528	0.528	0.520	0.310
R-squared	-	-	-	**0.667**	0.520	0.657	0.541	0.541	0.544	0.490
Bias	-	-	-	**2.853**	6.417	3.254	9.855	9.857	3.121	6.159

Note: The unit used is mmol/gDCW/hr. [1]Actual refers to the lab experiment results provided by the previous work (Shen et al., 2019). The substrate uptake rate was glucose 20 mmol/gDCW/hr in anaerobic conditions. [2] The values are provided by (Shen et al., 2019). Meanwhile, bold highlights refer to the best performance in the particular validation measure. The abbreviation of 'Met.', 'Bio.', 'Lit', 'Bd.', 'lb', and 'ub' refer to a metabolite, biomass percentage, literature, boundary, lower bound, and upper bound, respectively.

References

Shen, F., Sun, R., Yao, J., Li, J., Liu, Q., Price, N. D., Liu, C., and Wang, Z. (2019). OptRAM: In-silico strain design via integrative regulatory-metabolic network modeling. *PLOS Computational Biology*, 15(3):e1006835.

*
