# Supplementary material for: RBI: a novel algorithm for regulatory-metabolic network model in designing the optimal mutant strain
Source: PeerJ Comput Sci. 2025 May 27;11:e2880. doi: 10.7717/peerj-cs.2880 (PMC12199197; doi:10.7717/peerj-cs.2880)
Supplement: Supplemental Information 13 [file peerj-cs-11-2880-s013.pdf]

Pseudocode of the reliability-based integrating (RBI) algorithm

Input :

${}_{m}\mathbf{X}_{a}$ // The stoichiometric matrix.
$\mathbf{D}$ // The gene expression data.
$\mathbf{g}$ // Vector of the metabolic genes (MG).
$\mathbf{h}$ // Vector of the regulatory genes (RG).
$\mathbf{a}$ // Vector of the boolean equation in GRNs.
$\mathbf{b}$ // Vector of the boolean equation in GPR.
Parameters α, β, γ.

Output : v_{biomass} and $v_{\text{metabolite}}$

Process :

Step	Complexity
Determine k regulatory gene knocked out.	$O(1)$
Binarizing $\mathbf{D}$ based on α.	$O(n_1)$
for i : 1 to $n(h)$	$O(n_2)$
if RG_i is knocked out	
$p_{\text{RG}_i} \Leftarrow 0$	
else	
$p_{\text{RG}_i} \Leftarrow f(\text{RG}_i)$	
end	
end	
Compute r_{MG_j}	$O(n_1)$
Calculate r_{MR_k}	$O(n_3)$
Determine $[\text{lb}_k^*, \text{ub}_k^*]$ using FVA	$O(n_4.n_3)$
Generate $\tilde{\mathbf{v}}$ // It is a vector containing the metabolic reaction (MR) categorized as the transport reaction or the 'ATPM' reaction	$O(n_3)$
for k : 1 to a	$O(a)$
if ($r_{\text{MR}_k} < \beta$) or ($\text{MR}_k \notin \tilde{\mathbf{v}}$)	
$\text{ub}_k \Leftarrow r_{\text{MR}_k} \times \text{ub}_k^* + \gamma$	
$\text{lb}_k \Leftarrow r_{\text{MR}_k} \times \text{lb}_k^* - \gamma$	
end	
end	
Calculate v_{biomass} using FBA	$O(n_4)$
$\text{lb}_{\text{biomass}} \Leftarrow 0.1 \times v_{\text{biomass}}$	$O(1)$
Calculate $v_{\text{metabolite}}$ using FBA	$O(n_4)$

*
