# Supplementary material for: RBI: a novel algorithm for regulatory-metabolic network model in designing the optimal mutant strain
Source: PeerJ Comput Sci. 2025 May 27;11:e2880. doi: 10.7717/peerj-cs.2880 (PMC12199197; doi:10.7717/peerj-cs.2880)
Supplement: Supplemental Information 14 [file peerj-cs-11-2880-s014.pdf]

Details of strains used to compare RBI algorithm efficiency against others

**Strain**	**The number of**				**Reference**
	**Metabolites**	**Reactions**	**Genes**	**TFs**	
E. coli core	72	95	137	18	Orth et al. (2010)
iAF1260	1668	2382	1261	49	Feist et al. (2007)
iJO1366	1805	2583	1367	49	Orth et al. (2011)
iMM904	1226	1577	905	99	Mo et al. (2009)
iTO977	1218	1562	961	98	Osterlund et al. (2013)
Yeast7	2220	3498	910	99	Aung et al. (2013)

References

Orth, J. D., Fleming, R. M. T., and Palsson, B. O. (2010). Reconstruction and Use of Microbial Metabolic Networks: the Core *Escherichia coli* Metabolic Model as an Educational Guide. *EcoSal Plus*, 4(1):ecosalplus.10.2.1.

Feist, A. M., Henry, C. S., Reed, J. L., Krummenacker, M., Joyce, A. R., Karp, P. D., Broadbelt, L. J., Hatzimanikatis, V., and Palsson, B. O. (2007). A genome-scale metabolic reconstruction for escherichia coli k-12 mg1655 that accounts for 1260 orfs and thermodynamic information. *Molecular Systems Biology*, 3(1):121.

Orth, J. D., Conrad, T. M., Na, J., Lerman, J. A., Nam, H., Feist, A. M., and Palsson, B. O. (2011). A comprehensive genome-scale reconstruction of escherichia coli metabolism—2011. *Molecular Systems Biology*, 7(1):535.

Mo, M. L., Palsson, B. O., and Herrgard, M. J. (2009). Connecting extracellular metabolomic measurements to intracellular flux states in yeast. *BMC Systems Biology*, 3(1):37.

Osterlund, T., Nookaew, I., Bordel, S., and Nielsen, J. (2013). Mapping condition-dependent regulation of metabolism in yeast through genome-scale modeling. *BMC Systems Biology*, 7(1):36.

Aung, H. W., Henry, S. A., and Walker, L. P. (2013). Revising the Representation of Fatty Acid, Glycerolipid, and Glycerophospholipid Metabolism in the Consensus Model of Yeast Metabolism. *Industrial Biotechnology*, 9(4):215–228.

*
