# Supplementary material for: RBI: a novel algorithm for regulatory-metabolic network model in designing the optimal mutant strain
Source: PeerJ Comput Sci. 2025 May 27;11:e2880. doi: 10.7717/peerj-cs.2880 (PMC12199197; doi:10.7717/peerj-cs.2880)
Supplement: Supplemental Information 15 [file peerj-cs-11-2880-s015.pdf]

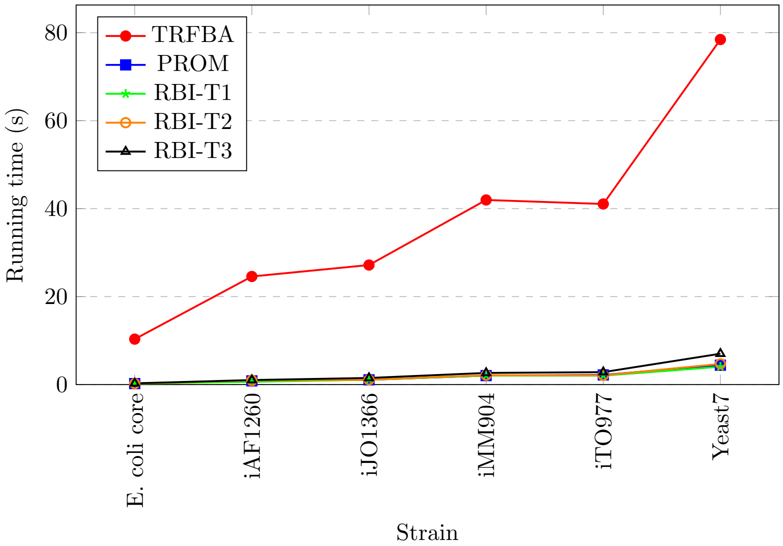

Graph depicting the running times of the RBI-T1, RBI-T2, RBI-T3, PROM, and TRFBA algorithms on a specific strain model.

*
