# Supplementary material for: RBI: a novel algorithm for regulatory-metabolic network model in designing the optimal mutant strain
Source: PeerJ Comput Sci. 2025 May 27;11:e2880. doi: 10.7717/peerj-cs.2880 (PMC12199197; doi:10.7717/peerj-cs.2880)
Supplement: Supplemental Information 16 [file peerj-cs-11-2880-s016.pdf]

The production rate of the succinate production

**Strain**	**Wild type**	**RBI-T1**	**RBI-T2**	**RBI-T3**	**PROM**	**TRFBA**
Aerobic						
E. coli core	9.601	9.598	9.537	9.577	9.601	**10.351**
iAF1260	12.854	12.944	**14.846**	13.440	12.854	11.901
iJO1366	13.076	13.121	13.485	**13.496**	13.076	13.076
Anaeobic						
E. coli core	30.210	30.420	30.628	**30.878**	30.130	30.841
iAF1260	32.505	32.722	**35.881**	34.961	32.406	35.655
iJO1366	34.423	34.325	34.840	**34.871**	34.325	34.325

Note: The unit used is mmol/gDCW/hr.

*
