# Supplementary material for: RBI: a novel algorithm for regulatory-metabolic network model in designing the optimal mutant strain
Source: PeerJ Comput Sci. 2025 May 27;11:e2880. doi: 10.7717/peerj-cs.2880 (PMC12199197; doi:10.7717/peerj-cs.2880)
Supplement: Supplemental Information 17 [file peerj-cs-11-2880-s017.pdf]

The production rate of the ethanol production

Strain	Wild type	RBI-T1	RBI-T2	RBI-T3	PROM	TRFBA
Aerobic						
iMM904	**11.348**	11.064	11.323	11.049	**11.348**	**11.348**
iTO977	13.923	13.848	12.615	**14.064**	13.298	13.298
Yeast 7.6	**10.920**	**10.920**	**10.920**	**10.920**	**10.920**	**10.920**
Anaerobic						
iMM904	41.016	39.547	40.916	40.558	**41.513**	**41.513**
iTO977	41.055	43.477	42.033	**43.554**	43.130	43.126
Yeast 7.6	39.826	39.374	**40.741**	39.503	40.517	39.588

Note: The unit used is mmol/gDCW/hr.

*
