# Supplementary material for: RBI: a novel algorithm for regulatory-metabolic network model in designing the optimal mutant strain
Source: PeerJ Comput Sci. 2025 May 27;11:e2880. doi: 10.7717/peerj-cs.2880 (PMC12199197; doi:10.7717/peerj-cs.2880)
Supplement: Supplemental Information 18 [file peerj-cs-11-2880-s018.pdf]

Schemes of the optimal mutant strains obtained by the RBI algorithm

Strain	Cond.	Algorithm	GR	PR	TF-KO schemes
Succinate production					
E.coli core	AER	RBI-T1	0.642	9.598	arca, phob, iclr, glcc, fnr
	ANA	RBI-T3	0.522	**30.878**	nac, fnr, pdhr, arca, glcc
iAF1260	AER	RBI-T2	0.924	**14.846**	purr, fis, uxur, oxyr, lrp
	ANA	RBI-T2	0.966	**35.881**	iclr, fis, pdhr, rob, narl
iJO1366	AER	RBI-T3	0.871	**13.496**	mara, gcva, fis, oxyr, fur
	ANA	RBI-T3	0.637	**34.871**	argr, oxyr, fur, gcvr, iclr
Ethanol production					
iMM904	AER	RBI-T2	0.663	11.323	thi2, arg81, mig2, mot3, ppr1
	ANA	RBI-T2	0.560	40.916	gln3, hap1, cha4, met4, arg80
iTO977	AER	RBI-T3	0.617	**14.064**	gcn4, cha4, upc2, met32, adr1
	ANA	RBI-T3	0.832	**43.554**	nrg1, pho4, mth1, cat8, gcn4
Yeast7.6	AER	All type	0.855	10.920	stp2, dal80, ino2, stp1, cat8
	ANA	RBI-T2	0.605	**40.741**	hap2, ino2, sip4, mig1, cat8

Note: The unit used is mmol/gDCW/hr. AER and ANA refer to aerobic and anaerobic, respectively.

*
